# Applications of Artificial Intelligence in Psychiatry and Psychology Education: Scoping Review

**DOI:** 10.2196/75238

**Published:** 2025-07-28

**Authors:** Julien Prégent, Van-Han-Alex Chung, Inès El Adib, Marie Désilets, Alexandre Hudon

**Affiliations:** 1 Department of Psychiatry and Addictology Faculty of Medicine Université de Montréal Montreal, QC Canada; 2 Faculty of Medicine McGill University Montreal, QC Canada; 3 Department of Psychiatry Institut universitaire en santé mentale de Montréal Montréal, QC Canada; 4 Department of Psychiatry Institut national de psychiatrie légale Philippe-Pinel Montreal, QC Canada; 5 Centre de recherche de l'Institut universitaire en santé mentale de Montréal Montreal, QC Canada

**Keywords:** artificial intelligence, medical education, psychiatry education, psychology education, machine learning, digital health, chatbot, clinical decision support, e-learning, mental health training

## Abstract

**Background:**

Artificial intelligence (AI) is increasingly integrated into health care, including psychiatry and psychology. In educational contexts, AI offers new possibilities for enhancing clinical reasoning, personalizing content delivery, and supporting professional development. Despite this emerging interest, a comprehensive understanding of how AI is currently used in mental health education, and the challenges associated with its adoption, remains limited.

**Objective:**

This scoping review aimed to identify and characterize current applications of AI in the teaching and learning of psychiatry and psychology. It also sought to document reported facilitators of and barriers to the integration of AI within educational contexts.

**Methods:**

A systematic search was conducted across 6 electronic databases (MEDLINE, PubMed, Embase, PsycINFO, EBM Reviews, and Google Scholar) from inception to October 2024. The review followed Preferred Reporting Items for Systematic Reviews and Meta-Analyses Extension for Scoping Reviews (PRISMA-ScR) guidelines. Studies were included if they focused on psychiatry or psychology, described the use of an AI tool, and discussed at least 1 facilitator of or barrier to its use in education. Data were extracted on study characteristics, population, AI application, educational outcomes, facilitators, and barriers. Study quality was appraised using several design-appropriate tools.

**Results:**

From 6219 records, 10 (0.2%) studies met the inclusion criteria. Eight categories of AI applications were identified: clinical decision support, educational content creation, therapeutic tools and mental health monitoring, administrative and research assistance, natural language processing (NLP), program/policy development, students’ study aid, and professional development. Key facilitators included the availability of AI tools, positive learner attitudes, digital infrastructure, and time-saving features. Barriers included limited AI training, ethical concerns, lack of digital literacy, algorithmic opacity, and insufficient curricular integration. The overall methodological quality of included studies was moderate to high.

**Conclusions:**

AI is being used across a range of educational functions in psychiatry and psychology, from clinical training to assessment and administrative support. Although the potential for enhancing learning outcomes is clear, its successful integration requires addressing ethical, technical, and pedagogical barriers. Future efforts should focus on AI literacy, faculty development, and institutional policies to guide responsible and effective use. This review underscores the importance of interdisciplinary collaboration to ensure the safe, equitable, and meaningful adoption of AI in mental health education.

## Introduction

Cloud computing, artificial intelligence (AI), machine learning (ML), telehealth, digitally assisted diagnosis and treatment, and consumer-focused mobile health applications have reshaped the landscape of health care delivery. These technologies are now widely used in clinical care, scientific research, and self-management [1]. These developments offer the potential to improve treatment outcomes, promote greater patient engagement, and enable earlier diagnosis and intervention [1]. In addition to enhancing conventional clinical procedures, such as teleconsultation and patient record management, these advancements have made way for fresh, data-driven methods of diagnosing and treating patients with a wide range of illnesses [2]. Particularly in the fields of psychology and psychiatry, new research highlights the expanding impact of AI online learning environments, and e-therapies, all of which could potentially reshape clinical practice, educational routes, and health policy [3,4]. As they provide new avenues for psychiatric condition screening, diagnosis, and monitoring, AI and ML have, in fact, attracted a lot of attention in the field of mental health care [3]. Self-guided mental health apps and web-based cognitive behavioral therapy (CBT) modules are examples of digital interventions that can help address systemic gaps in mental health care by improving access in underserved areas, reducing stigma, and lowering infrastructure costs. Still, limitations, such as weakened therapeutic alliance, limited support for complex conditions, and digital literacy barriers persist, highlighting the need for complementary innovations, such as AI [5]. The goal of ML, a branch of AI, is to empower computers to learn from data and make predictions or judgments by using statistical models and algorithms [6]. Potential applications of ML include predicting the effectiveness of antidepressant medicine, defining depression, estimating the risk of suicide, and predicting psychotic episodes in people with schizophrenia [7-14]. Beyond individual diagnosis and prognosis, ML has also been explored for system-level uses, such as optimizing service triage, forecasting population mental health trends, and informing resource allocation strategies [3]. Although promising, these uses raise critical concerns about the limits of algorithmic decision-making in capturing complex human experiences, therapeutic nuance, and clinical judgment.

In any case, any increase in accuracy or efficiency must be balanced against possible drawbacks, including algorithmic bias, concerns about data privacy, and the requirement for open systems to maintain patient confidence [15]. There are concerns over chatbots and other automated therapy tools’ ability to give compassionate care and uphold therapeutic boundaries, despite being promoted as affordable ways to provide mental health help [16]. Therefore, the use of AI-based technologies (eg, chatbots, virtual assistants, or automated therapy platforms) necessitates careful evaluation of any potential negative effects, such as algorithmic bias, transparency issues, patient confidentiality, and wider ethical issues [16,17]. These tools are already transforming the patient-clinician relationship and raise complex questions about how therapeutic alliances can be established and maintained in digital environments [18]. The traditional roles and responsibilities of health care providers may change because of the advent of automated evaluations and AI-driven therapy recommendations. As a matter of fact, clinicians will soon have to balance the results of algorithms with their professional judgment [2].

These factors highlight the need for careful consideration for psychologists, psychiatrists, and other medical practitioners to gain a thorough understanding of the ethical and therapeutic aspects of developing technology [19]. Numerous experts stress that substantial training for aspiring professionals is necessary for a meaningful integration of AI into mental health treatment, especially when it comes to e-therapy techniques or semiautomated diagnostics [19,20]. Beyond the purview of one’s initial licensing, lifelong learning in order to keep up with technological advancements to guarantee that mental health practitioners stay knowledgeable about the rapidly changing field of e-therapies and digital health solutions could be seen as nonnegotiable [21]. This need aligns with constructivist and experiential learning theories, which emphasize that learners build knowledge most effectively through active engagement and real-world application, both of which are necessary for the safe and ethical use of AI in clinical practice. The requirements for certification and continuing education programs, which may include specific workshops, learning modules centered on digital proficiency, or periodic re-examinations, reflect these needs [22]. Education researchers warn that lectures or brief seminars alone will not be sufficient to close the knowledge gap; clinical and research experiences that enable practitioners to assess, adjust, and use AI technologies with confidence in a safe and ethical way are also necessary [23].

The purpose of this scoping review was to determine the types of AI that are currently used in academic programs and educational curricula in psychology and psychiatry. Furthermore, it attempted to identify the barriers to and facilitators of such uses. By evaluating various apps, this review aimed to synthesize existing apps and highlight areas where further development or inquiry may be warranted. These included not only content delivery and assessment but also more challenging domains, such as teaching clinical judgment, fostering empathy, assessing nuanced interpersonal interactions, and supporting therapeutic decision-making—areas that are often context dependent and difficult to replicate algorithmically. Finally, suggestions were offered for future research directions to support the responsible and effective incorporation of AI into mental health education.

## Methods

### Search Strategies

A broad scoping search was systematically performed to retrieve the recent literature from multiple electronic databases, including Medline, PubMed, Embase, PsycNet (PsycINFO), EBM Reviews – Cochrane Database of Systematic Reviews, and Google Scholar, covering records from database inception through October 2024. The review adhered to the Preferred Reporting Items for Systematic Reviews and Meta-Analyses Extension for Scoping Reviews (PRISMA-ScR) guidelines [24]. The search strategy integrated both free-text keywords and controlled vocabulary (Medical Subject Headings [MeSH] terms), centering on themes related to AI (eg, “artificial intelligence,” “AI”) and medical education (eg, “education,” “medical,” “students”), in accordance with the study’s aims. Full search strategies are detailed in Multimedia Appendix 1. The search strategies were designed by an experienced librarian in the field of mental health (author MD). They were also cross-validated by another librarian using the Peer Review of Electronic Search Strategies (PRESS) approach. The methodology was designed by the corresponding author; searches were conducted by author AH and independently verified by author JP. No filters were applied concerning geographical location or institutional setting. The completed PRISMA-ScR checklist is available in Multimedia Appendix 2.

### Study Eligibility

Studies were eligible for inclusion if they met the following criteria: (1) study focused on a topic within the fields of psychiatry or psychology education; (2) involved the use of an AI tool, model, or approach; (3) included a discussion of facilitators of or barriers to the use of AI; and (4) were available in either English or French. Papers that did not pertain to psychiatry or psychology or that referenced AI technologies without a clearly defined implementation or application were excluded. In addition, studies were excluded if they featured AI tools outside the domain of relevance, such as rule-based expert systems or search algorithms unrelated to data-driven models. Unpublished studies and gray literature were not considered for inclusion.

### Data Extraction

A standardized data extraction form was developed to systematically collect and organize relevant information from each included study. Data extraction was performed independently by at least 2 of the authors, with discrepancies resolved through discussion and consensus or by consulting a third author, when necessary. The process was guided by the research objectives for examining the integration of AI in health profession education.

The following elements were extracted from each study:

- Authors: citation details, including the first author and year of publication.
- Population: description of the study participants, including the sample size, educational level (eg, undergraduate, postgraduate, continuing professional development), and discipline (eg, psychiatry, psychology, medical education).
- Use of AI: specific application or role of AI within the educational context, such as adaptive learning platforms, natural language processing (NLP) tools, intelligent tutoring systems, virtual patient simulations, or predictive analytics.
- Main outcomes: primary findings related to educational effectiveness, learner satisfaction, knowledge acquisition, clinical reasoning, engagement, or other measured outcomes. Where applicable, outcomes were categorized by study design (qualitative, quantitative, or mixed methods).
- Facilitators: factors that supported the successful implementation or perceived value of AI-enhanced educational interventions, such as user-friendliness, institutional support, personalization of learning, or alignment with curricular goals.
- Barriers: reported challenges or limitations in the adoption of AI tools, including technical constraints, ethical concerns, paucity of digital literacy among users, limited evidence of effectiveness, or resistance to change within educational environments.

Extracted data were tabulated in Microsoft Excel (version 17.0) to allow comparison across studies and to identify patterns, gaps, and emerging themes related to the integration of AI in teaching and learning within the fields of psychiatry and psychology.

### Quality Assessment

To examine the included studies’ methodology, clarity, and transparency, we carried out a systematic evaluation. Considering the variety of research designs seen in the literature on AI in psychiatry and addiction education, this phase attempted to improve the findings’ interpretability. We used evaluation instruments that were specific to each research type’s design to guarantee methodological adequacy.

The Joanna Briggs Institute (JBI) Checklist for Analytical Cross-Sectional Studies was used to assess both quantitative and observational research [25]. The methodological soundness of studies looking at correlations between exposures and outcomes at a specific moment in time can be evaluated with this tool. Items on the checklist evaluate the appropriateness of statistical analyses, the identification and control of confounding factors, the validity and reliability of exposure and outcome measurements, and the clarity of the inclusion criteria.

The JBI Checklist for Qualitative Research was also used [25]. This tool assesses how well research methodology and research topics align, how data are collected, how participant voices are represented and interpreted, and how much the researcher has influenced the study. To guarantee that qualitative insights are obtained through a thorough and reliable process, it also evaluates ethical issues and the openness of data analysis techniques.

Mixed methods studies were appraised using the Mixed Methods Appraisal Tool (MMAT), 2018 version [26]. The MMAT is a validated tool specifically developed for the assessment of studies that combine qualitative and quantitative approaches. It allows for concurrent evaluation of the components of each methodology within a single study and includes criteria for the integration of qualitative and quantitative data, the appropriateness of the design to the research questions, and the coherence of interpretations drawn from the combined methods.

For nonempirical contributions, such as viewpoints, editorials, and conceptual papers, we used the authority, accuracy, coverage, objectivity, date, and significance (AACODS) checklist [27]. This framework assesses 6 key domains: authority (credibility of the author or source), accuracy (evidence supporting claims), coverage (comprehensiveness of content), objectivity (balance and absence of bias), date (currency and relevance), and significance (contribution to the field). It is particularly useful for evaluating gray literature and opinion-based texts where conventional empirical criteria may not apply.

All studies were reviewed independently by 2 researchers (JP and AH). Each appraisal tool includes a set of key domains that were rated as “yes,” “partial,” or “no” based on the extent to which the methodological criteria were clearly addressed and appropriately implemented in each study.

## Results

### Description of Studies

**Figure 1:**
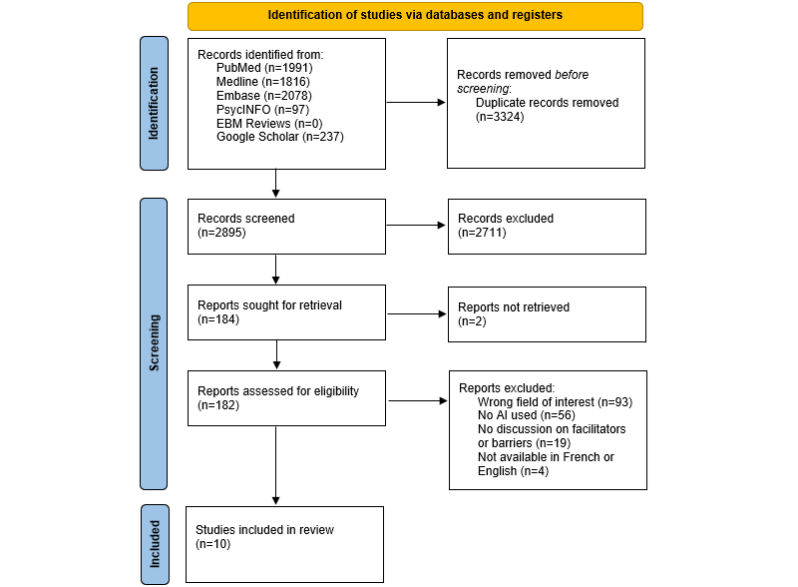
PRISMA-ScR flowchart for the inclusion of studies.

The scoping review explored the use of AI in teaching and learning within psychiatry and psychology. The initial search across 6 electronic databases yielded 6219 records. After removing 3324 (53.4%) duplicates, 2895 (46.6%) records were screened by title and abstract. Of these, 2711 (93.6%) papers were excluded for not meeting the inclusion criteria. A total of 184 (6.4%) full-text papers were sought for retrieval, with 2 (1.1%) reports not successfully retrieved. The remaining 182 (98.9%) papers were assessed for eligibility in full. Following detailed evaluation, 172 (94.5%) papers were excluded due to being outside the field of interest (n=93, 54.1%), lacking the use of AI (n=56, 32.6%), not addressing facilitators or barriers (n=19, 11%), or not being available in English or French (n=4, 2.3%). Ultimately, 10 (5.5%) studies met all inclusion criteria and were included in the final analysis. A flowchart summarizing the selection process is presented in Figure 1, and details of the included studies can be found in Multimedia Appendix 3.

### Main Uses of Artificial Intelligence

Across the 10 studies [28-37] included in this scoping review, AI was applied to a variety of educational functions in psychiatry and psychology. The most frequently observed category was *clinical decision support* (n=5, 50%), where AI tools were used to train learners in diagnosis, prognosis, risk assessment, and early intervention strategies. This was followed by 5 categories that each appeared in 3 (30%) studies: *educational content creation and enhancement*, *therapeutic tools and mental health monitoring*, *administrative and research assistance*, *NLP applications*, and *program/policy development*. These categories reflect AI’s growing role in the development of educational materials, therapeutic simulations, and institutional planning. Less frequently, studies addressed AI’s applications in *professional development and assessment* (n=1, 10%) and *student/applicant support* (n=1, 10%), highlighting emerging but less explored domains. This distribution suggests a strong current emphasis on clinical reasoning, content automation, and digital service integration in educational contexts.

#### Clinical Decision Support

AI tools are increasingly integrated into psychiatry and psychology education to train learners in diagnosis, prognosis, and risk assessment. Through exposure to AI-driven systems, such as ML models and NLP tools, trainees can learn how to identify suicide risk, detect patterns in substance use disorders, or evaluate the progression of neurodegenerative conditions [28]. Banerjee et al [29] emphasized the importance of training programs that illustrate how AI can triage patients or generate diagnostic suggestions, helping doctors understand both the power and limitations of these technologies. Furthermore, students are being introduced to AI’s role in treatment personalization, such as adapting therapy plans or medication regimens using predictive modeling [30]. These systems also facilitate early intervention, teaching clinicians how AI can flag high-risk patients in real time, thus embedding preventive care principles into training [31].

#### Educational Content Creation and Enhancement

Generative AI is transforming the design and delivery of educational content in psychiatry and psychology. One example is the use of ChatGPT to develop script concordance tests (SCTs), which promote clinical reasoning in undergraduate medical education. Hudon et al [32] demonstrated that AI-generated SCTs are nearly indistinguishable from those written by human experts, highlighting AI’s potential to rapidly generate quality training materials aligned with psychiatric diagnostic frameworks. In addition, AI can support exam preparation, providing structured explanations, study plans, and interactive feedback for licensing exams and clinical cases [28]. Spallek et al [31] noted that although AI tools require human oversight, they offer accessible and customizable resources that align with the best practices in mental health communication and literacy, ultimately empowering educators and enhancing learner engagement.

#### Student/Applicant Support

AI can now be used to assist learners with the residency and fellowship application process, offering new educational opportunities in self-presentation and professional writing. Mangold and Ream [33] explored how students and faculty use AI to draft and refine personal statements and letters of recommendation, improving clarity and grammar and reducing biased language. From a pedagogical perspective, this practice teaches students to reflect critically on AI-generated outputs and engage in iterative editing processes. Moreover, AI is being proposed for application screening, potentially reducing human bias and increasing efficiency in admissions, which prompts institutions to educate both applicants and reviewers on ethical and equitable AI use [33]. These developments signal a shift in how learners engage with professional identity formation and how educators must adapt guidance accordingly.

#### Therapeutic Tools and Mental Health Monitoring

A body of literature highlighted AI’s use in therapeutic education, particularly around digital interventions, such as CBT- or acceptance and commitment therapy (ACT)-based apps and chatbots. Blease et al [30] and Gratzer and Goldbloom [34] emphasized the need to train future psychiatrists to evaluate and potentially integrate these tools into treatment plans. E-therapy technologies, including AI-powered chatbots, provide psychoeducation on demand and demonstrate how therapy can be delivered asynchronously [34]. Trainees also learn about real-time symptom tracking, which is increasingly used in digital platforms to monitor patient well-being and guide interventions. These digital tools expose students to new care modalities, challenging them to assess efficacy, ethics, and clinical utility in both individual and population-based care models [34].

#### Administrative and Research Assistance

AI is also reshaping how clinicians and students interact with administrative and scholarly tasks, including documentation, summarization, and literature reviews. Banerjee et al [38] observed that AI is viewed as helpful in reducing the time spent on clinical documentation, allowing more focus on direct learning and patient care. Additionally, AI tools are being used in academic psychiatry to assist with automated literature searches, summarization, and even initial drafting of manuscripts, offering educational insights into how information is synthesized and presented in research [28]. These uses favorize critical thinking and teach students to evaluate AI-generated content, thereby strengthening their roles as both users and producers of scientific knowledge.

#### Professional Development and Assessment

In postgraduate and continuing education contexts, AI supports competency-based assessment and professional development. Anzia [35] discussed how longitudinal assessment platforms, potentially enhanced by AI, are replacing traditional high-stakes exams in psychiatry, promoting lifelong learning and more reflective practice. These tools can track learning progress, provide feedback, and recommend tailored educational pathways. Moreover, AI may be integrated into clinical skills evaluations, simulating patient scenarios or providing automated assessments of diagnostic reasoning. This shift calls for educators to incorporate digital literacy and AI fluency into curricula, ensuring that learners are prepared to navigate evolving certification and assessment landscapes [35].

#### Natural Language Processing Applications

NLP tools powered by AI are being introduced in educational settings to illustrate how clinical language can be analyzed, interpreted, and used for real-time support. Banerjee et al [29] noted NLP’s utility in automating clinical documentation, reducing the clerical burden and highlighting the importance of clear, structured input. In psychiatry training, NLP is also applied to suicide risk detection, teaching learners how digital footprints and language cues from online interactions can signal crisis [28]. Additionally, NLP technologies are integrated into telehealth platforms, enhancing communication between patients and providers, and offering opportunities for students to learn how AI can support culturally sensitive, evidence-based interactions [31].

#### Program/Policy Development

Finally, AI’s influence on institutional teaching structures is growing, particularly through the design of AI-compatible teaching modules and the creation of policies to guide AI use. Manjunatha et al [36] illustrated how telepsychiatry programs in India are incorporating AI into remote learning for primary care doctors, serving as a scalable model for underserved settings. These modules reflect a shift toward digital curricula that embed clinical translation and evidence-based AI use. Similarly, Mangold and Ream [33] emphasized the need for training programs to define guidelines for AI use in admissions and evaluation, prompting educators to prepare learners for ethical dilemmas and policy engagement in the evolving digital landscape.

### Facilitators

A number of facilitators support the integration of AI into teaching and learning in psychiatry and psychology.

#### Technological Readiness and Tool Availability

A recurring theme across the 10 studies was the availability and accessibility of AI tools, particularly large language models, such as ChatGPT, which simplify the creation of educational content and customization of learning materials for diverse learner needs [31,32]. These tools are perceived as valuable due to their ability to generate structured and accessible outputs, supporting educators in preparing mental health scenarios or assessments rapidly and effectively. Moreover, other technologies, such as telepsychiatry and mobile-based learning platforms, are supported by the widespread use of smartphones and digital infrastructure, increasing scalability and access to remote or underserved areas [36].

#### Educational and Efficiency Enhancement

Several studies have emphasized that structured prompting or prompt engineering significantly enhances output quality, improving both relevance and accuracy for educational use [33]. In clinical training contexts, AI is seen as a time-saving facilitator, capable of reducing administrative burdens, such as documentation, and allowing learners to focus on clinical reasoning and decision-making [29]. In addition, generative AI technologies and related models can facilitate the production of highly realistic synthetic data and the seamless integration of unstructured content across diverse formats [37]. These innovations have the potential to transform core practices, such as risk assessment, diagnostic decision-making, and treatment planning, while simultaneously creating new opportunities in educational and training environments.

#### Learner Engagement and Openness

Finally, positive attitudes from students and trainees, particularly their willingness to explore new tools, and their recognition of AI’s role in increasing efficiency, accuracy, and engagement, are essential to AI adoption in educational environments [30]. These facilitators reflect a confluence of technological readiness, user engagement, and curricular flexibility.

### Barriers

Despite growing enthusiasm, several barriers hinder the seamless integration of AI into psychiatry and psychology education.

#### Digital and Educational Gaps

A dominant concern is the absence of formal training and digital literacy among students and educators, which limits their ability to interpret and critically evaluate AI-generated outputs [29,30]. Many studies have noted a limited presence of AI content in medical curricula, resulting in missed opportunities to prepare learners for evolving clinical environments where AI plays a central role [30].

#### Ethical and Legal Issues

There are also ethical and legal concerns, particularly around data privacy, algorithmic bias, and informed consent, which raise questions about the responsible use of AI tools, such as chatbots or diagnostic aids [31,34]. The scarcity of high-quality data and the opacity of AI algorithms, often referred to as the “black box” problem, are also cited as major obstacles to trust and widespread adoption [28]. Additionally, concerns persist about the accuracy and reliability of AI-generated educational materials, particularly when outputs are not subject to expert review, posing risks of misinformation or oversimplification [31,32].

#### Pedagogical Limitations

Finally, several papers highlighted that AI outputs can lack nuance, empathy, or personalization, making them less suitable for teaching relational and humanistic aspects of psychiatric care [33].

These barriers highlight the need for comprehensive strategies that include AI literacy, ethical guidance, and faculty support to ensure safe and effective integration into educational practice.

### Quality Assessment of the Identified Studies

Overall, the methodological quality of the included literature was moderate to high. Mixed methods and empirical studies demonstrated clear objectives, coherent data collection and analysis strategies, and ethical transparency. However, several studies lacked detailed descriptions of sampling procedures or integration of data types. Nonempirical papers were generally strong in authority and relevance but limited by the absence of primary data or systematic methodology. Despite variability in design and depth, most studies provided valuable insights into the educational applications of AI in psychiatry and psychology. The full quality appraisal is presented in Multimedia Appendix 3.

## Discussion

### Principal Findings

This scoping review aimed to identify the different ways AI is currently used in the teaching and learning of psychiatry and psychology. A total of 10 studies were fully analyzed, and 8 categories of AI applications were identified: clinical decision support, educational content creation and enhancement, therapeutic tools and mental health monitoring, administrative and research assistance, NLP applications, program and policy development, student/applicant support, and professional development and assessment. These categories reflect the diverse roles AI plays in shaping educational strategies, curricular design, and learner engagement in mental health training. The studies included were overall of moderate-to-high quality. The most notable facilitators to AI integration in teaching and learning in psychiatry and psychology are technological readiness and tool availability, educational and efficiency enhancement, and learner engagement and openness. The barriers that hinder the integration of AI into psychiatry and psychology education are digital and educational gaps, ethical and legal issues, and pedagogical limitations.

### Comparison With Prior Work

The findings of this scoping review confirm and expand on other studies that demonstrate the growing integration of AI into psychological and psychiatric education, especially through clinical decision support technologies. Previous research has shown, for example, that ML models can help forecast the risk of depression, schizophrenia, and suicide. Our study also noted that similar predictive technologies are already being used in educational contexts [38,39]. This is consistent with the findings of Rajkomar et al [40], who observed that clinical AI tool exposure aids in the development of important data literacy in health care trainees. Our work showcases the potential of AI in supporting students as they develop their critical reasoning. However, it is important to keep in mind that these critical reasoning skills are still mostly shaped over time through clinical exposure and case discussion, both of which cannot be replaced by algorithms or AI. The multidisciplinary team also holds a significant place in clinical management and risk sharing. AI can give theoretical advice to students regarding how to lead a team, but it cannot teach a student how to lead a team in real time. Although previous research has often emphasized clinical outcomes, we focused here on the learning process itself, with the understanding that AI is only one of many educational tools.

The literature on AI also supports the increasing use of generative AI in the production of instructional materials. Recent research has switched to looking at how tools such as ChatGPT, GPT-4, and Claude might scaffold learning in medical education, whereas the majority of earlier applications were on patient-facing educational interventions (eg, mental health apps) [41]. These studies support our findings by demonstrating that AI is capable of creating excellent test questions, simulating clinical situations, and even co-creating course curricula. However, critical gaps still exist, notably the possibility that students will passively accept AI outputs without engaging sufficiently. In fact, some of these critical knowledge gaps might be due to the current state of research, which remains limited when it comes to assessing how heavy reliance on dialogue AI may affect decision-making, critical thinking, and analytical reasoning in both educational and research contexts [42]. Meskó [43] shared this concern and called for clear instruction in AI prompt engineering, appropriate medical professional tutorials, and verification skills. The findings of this scoping review highlight that such competencies are increasingly essential in psychiatry and psychology, where nuance and context matter deeply.

According to certain studies, an important area of AI integration in training is mental health apps and therapeutic chatbots, which is consistent with previous research [44,45]. According to these studies, chatbot-based psychotherapy and psychoeducational tools are beneficial teaching tools, in addition to being successful for patients. When included in clinical simulations, they give students an opportunity to analyze intervention results, gauge the therapeutic tone, and practice making moral decisions. Our results highlight the need for directed education to guarantee that students acquire abilities in digital empathy, data protection, and cultural adaptation, even when these tools show promise. Training programs need to change in a way that assists clinicians in interpreting AI-driven outputs, while upholding person-centered treatment and therapeutic alliances, as D’Alfonso et al [46] contended.

Lastly, the findings confirm that faculty development, institutional preparedness, and ethical advice are drivers of AI adoption, which is in line with research on digital transformation in health professions education [47]. Although students frequently use AI tools for convenience, educators are nevertheless worried about the loss of critical thinking, professional identity, and interpersonal skills. These difficulties point to the necessity of organized curricula, such as one that incorporates AI literacy into psychology and psychiatry undergraduate and graduate education programs [48].

### Directions for Further Research

This scoping review revealed that slowly but surely, the integration of AI, although remaining nascent in psychology and psychiatry education, is nonetheless there to stay. Future research should prioritize rigorous, outcome-based studies that evaluate further the impact of the AI-enhanced educational tools that this paper described, such as diagnostic stimulation, e-therapies, AI-assisted clinical decision-making, and the impact on real-life learning performance. Another important aspect that would be crucial to investigate is the management of algorithmic bias and transparency and understanding the extent of protecting one’s private data. Research that captures the perspectives of students and educators could shed light on readiness, perceived barriers, and opportunities for meaningful adoption of AI.

Furthermore, given the fact that the majority of studies analyzed are from Euro-American and high-income contexts, there is a clear need for research that centers on diverse populations, including Indigenous, racialized, and culturally distinct groups. Together, these directions can help guide the development of inclusive and ethically grounded approaches to AI integration into mental health education.

### Recommendations for Institutions

To ensure that psychology and psychiatry programs prepare trainees for a rapidly evolving clinical landscape, educational institutions should take proactive steps toward integrating AI literacy into core curricula. For example, digital literacy could be integrated early into medical education, with foundational topics such as ML and data ethics. This effort would benefit from interdisciplinary collaboration among health sciences, computer science, and bioethics departments to ensure a well-rounded approach.

The extent to which a person believes that a technology can enhance their performance at work (or enhance their learning experience, for that matter) is commonly referred to as “perceived usefulness,” and it plays a pivotal role in determining whether individuals are likely to adopt new technologies [49]. A meta-analysis by Scherer and Teo [50] identified perceived usefulness as a strong predictor of a teacher’s readiness to engage with digital tools. Conversely, barriers included anxiety and a lack of AI-specific training [51]. Thus, to effectively deal with the growing threats posed by AI, it seems crucial to promote the various uses of AI among the professors. Teachers who understand AI better are more equipped to use it in ways that meet the varied needs of their students [52]. In addition, when educators develop a solid understanding of AI, they are better equipped to tackle its ethical issues, such as algorithmic bias, data privacy, and the risk of becoming overly dependent on AI [53].

Hence, educators require adequate training, resources, and institutional support to effectively teach and oversee the responsible use of emerging digital tools in clinical and academic settings.

How will universities effectively manage the growing threats that AI presents to medical education? Those threats, as previously mentioned, include academic dishonesty in assessments (eg, plagiarism), the spread of misinformation, and the difficulty AI-using students face in discerning some of the nuances in fields where human interaction is key, such as psychiatry and psychology. Because these threats are often multifactorial in nature, their management requires an inclusive approach involving all key actors (students, educators, institutions) [54]. In practice, at the university level, an interesting avenue would be the constitution of a task force (or a committee) comprising students, faculty members, AI scholars, and IT personnel. This task force would be aimed at determining permissible uses of AI versus prohibited uses of AI and how to detect the latter. The detection of AI-generated content can be facilitated by applications such as GPTZero and QuillBot, for example. Higher education institutions (HEI) should not only establish such thorough guidelines but also review them periodically to ensure their continued relevance [54]. Bozkurt [55] suggested that HEI also require transparent disclosure of AI usage by their personnel and students (eg, in a given assessment, the students would be expected to explicitly declare the sections drafted by ChatGPT with human oversight). HEI could also offer some new job positions focused on AI or hire professionals specifically trained to identify AI-related academic misconduct and sanction such ethical issues. These professionals would ideally also have some degree of experience with the efficient implementation of AI in an academic context.

### Strengths and Limitations

The authors brought diverse disciplinary perspectives to this review, including training in psychology, psychiatry, medical education, and digital health. These backgrounds informed the framing of the review and the interpretation of its findings.

This scoping review also has a few limitations. First, although a comprehensive search strategy was used across multiple databases, it is possible that relevant studies were missing, particularly those published in nonindexed journals or categorized under broader terms not captured by the search keywords. Second, the authors acknowledge the potential for disciplinary bias rooted in Western academic systems. An effort was made to include literature from a range of geographic regions and institutional contexts; however, due to the limited published research available on the subject, the review was limited to studies published in English or French, potentially excluding valuable insights from non-English/French literature; in addition, the majority of published research available in English originated from Euro-American or high-income settings, which may limit the generalizability of the review’s conclusions. Third, due to the heterogeneity of study designs and the inclusion of both empirical and conceptual papers, no formal meta-analysis was conducted, and the synthesis remained primarily narrative. In addition, although efforts were made to assess study quality using validated tools, some included papers, particularly perspectives and editorials, lacked sufficient methodological detail, which may limit the generalizability of their conclusions. Finally, because the field of AI in mental health education is rapidly evolving, newer studies and technologies may have emerged since the time of data collection, warranting future updates to this review.

### Conclusion

To conclude, this scoping review provided an overview of how AI is being integrated into the teaching and learning of psychiatry and psychology. By analyzing 10 studies, 8 distinct categories of AI uses were identified, ranging from clinical decision support and educational content generation to digital therapeutic tools and policy development. These findings highlight the emerging role of AI not only as a clinical adjunct but also as a transformative educational tool that can support adaptive learning, promote efficiency, and broaden access to mental health training. Although the overall quality of the studies included was moderate to high, important challenges remain, particularly related to ethical considerations, digital literacy, and institutional readiness. As AI technologies continue to evolve, future research and curriculum development efforts should focus on promoting safe, equitable, and pedagogically sound integration of AI in mental health education. Equipping educators and students will require the integration of AI literacy into core curricula, embedding foundational topics such as ML and data ethics and fostering interdisciplinary collaboration between various departments. Faculty personnel development, institutional preparedness, and student involvement are essential drivers for successful AI adoption. Future research should prioritize rigorous, outcome-based studies and capture diverse perspectives in order to guide the development of inclusive and effective AI integration strategies in mental health education. This review underscores the need for ongoing interdisciplinary collaboration between educators, clinicians, technologists, and policymakers to ensure that future practitioners are well equipped to engage with AI in meaningful and responsible ways.

## Appendix

Multimedia Appendix 1 Electronic search strategy for the scoping review conducted.

Multimedia Appendix 2 PRISMA-ScR checklist.

Multimedia Appendix 3 Scoping review study selection detailed results and quality assessment.

## Abbreviations

AI: artificial intelligence
CBT: cognitive behavioral therapy
HEI: higher education institutions
JBI: Joanna Briggs Institute
ML: machine learning
MMAT: Mixed Methods Appraisal Tool
NLP: natural language processing
PRISMA-ScR: Preferred Reporting Items for Systematic Reviews and Meta-Analyses Extension for Scoping Reviews
SCT: script concordance test
