# Supplementary material for: Applications of Artificial Intelligence in Psychiatry and Psychology Education: Scoping Review
Source: JMIR Med Educ. 2025 Jul 28;11:e75238. doi: 10.2196/75238 (PMC12340458; doi:10.2196/75238)
Supplement: Multimedia Appendix 1 [file mededu_v11i1e75238_app1.docx]

**Multimedia Appendix 1.** Electronic search strategy for the scoping review conducted.

This supplementary material has been provided by the authors to give readers additional information about their work.

**Table S1. Electronic search strategy for the scoping review conducted.**

| **Database; Search** | **Search Terms** |
| --- | --- |
|  |  |
| PubMed; k= 1991 | ("Artificial Intelligence"[Mesh :NoExp] OR "artificial intelligence"[TIAB] OR AI[TIAB] OR Chatbot*[TIAB] OR "Conversational robot"[TIAB:~3] OR "conversational robots"[TIAB:~3] OR "Conversational agent"[TIAB:~3] OR "conversational agents"[TIAB:~3] OR "Digital agent"[TIAB:~3] OR "digital agents"[TIAB:~3] OR ChatGPT[TIAB] OR Gemini[TIAB]) AND ("Education, Medical"[Mesh:NoExp] OR "Education, Medical, Graduate"[Mesh] OR "Students, Medical"[Mesh] OR "Schools, Medical"[Mesh] OR "medical education"[TIAB:~3] OR "medical training"[TIAB:~3] OR "medical school"[TIAB:~3] OR "medical schools"[TIAB:~3] OR "medical curriculum"[TIAB:~3] OR "medical curriculums"[TIAB:~3] OR "medical curricula"[TIAB:~3] OR "medical curriculas"[TIAB:~3] OR "medical teaching"[TIAB:~3] OR "medical program"[TIAB:~3] OR "medical programs"[TIAB:~3] OR "medicine education"[TIAB:~3] OR "medicine training"[TIAB:~3] OR "medicine school"[TIAB:~3] OR "medicine schools"[TIAB:~3] OR "medicine curriculum"[TIAB:~3] OR "medicine curriculums"[TIAB:~3] OR "medicine curricula"[TIAB:~3] OR "medicine curriculas"[TIAB:~3] OR "medicine teaching"[TIAB:~3] OR "medicine program"[TIAB:~3] OR "medicine programs"[TIAB:~3] OR "resident education"[TIAB:~3] OR "residents education"[TIAB:~3] OR "resident training"[TIAB:~3] OR "residents training"[TIAB:~3] OR "resident teaching"[TIAB:~3] OR "residents teaching"[TIAB:~3] OR "resident learning"[TIAB:~3] OR "residents learning"[TIAB:~3] OR "medical student education"[TIAB:~3] OR "medical students education"[TIAB:~3] OR "medical student training"[TIAB:~3] OR "medical students training"[TIAB:~3] OR "medical student teaching"[TIAB:~3] OR "medical students teaching"[TIAB:~3] OR "medical student learning"[TIAB:~3] OR "medical students learning"[TIAB:~3] OR "medical trainee education"[TIAB:~3] OR "medical trainees education"[TIAB:~3] OR "medical trainee training"[TIAB:~3] OR "medical trainees training"[TIAB:~3] OR "medical trainee teaching"[TIAB:~3] OR "medical trainees teaching"[TIAB:~3] OR "medical trainee learning"[TIAB:~3] OR "medical trainees learning"[TIAB:~3] OR residency[TIAB] OR "fellowship program*"[TIAB]) |
| Medline; k= 1816 | 1. Artificial Intelligence/  2. ("artificial intelligence" or AI or Chatbot* or (conversational adj3 (robot* or agent*)) or (digital adj3 agent*) or ChatGPT or Gemini).ti,ab,kw.  3. or/1-2  4. Education, Medical/  5. exp Education, Medical, Graduate/  6. Students, Medical/  7. Schools, Medical/  8. ((medic* adj3 (education or training or school* or curricul* or teaching or program*)) or ((resident* or "medical student*" or "medical trainee*") adj3 (education or training or teaching or learning)) or residency or "fellowship program*").ti,ab,kw.  9. or/4-8  10. and/3,9 |
| Embase; k = 2078 | 1. artificial intelligence/  2. chatbot/ or exp artificial intelligence chatbot/  3. ("artificial intelligence" or AI or Chatbot* or (conversational adj3 (robot* or agent*)) or (digital adj3 agent*) or ChatGPT or Gemini).ti,ab,kw.  4. or/1-3  5. medical education/ or medical school/ or residency education/  6. exp medical student/  7. ((medic* adj3 (education or training or school* or curricul* or teaching or program*)) or ((resident* or "medical student*" or "medical trainee*") adj3 (education or training or teaching or learning)) or residency or "fellowship program*").ti,ab,kw.  8. or/5-7  9. and/4,8  10. limit 9 to (article or article in press or "preprint (unpublished, non-peer reviewed)" or "review") |
| PsycINFO; k = 97 | 1. artificial intelligence/  2. exp conversational agents/  3. generative artificial intelligence/  4. ("artificial intelligence" or AI or Chatbot* or (conversational adj3 (robot* or agent*)) or (digital adj3 agent*) or ChatGPT or Gemini).ti,ab,id.  5. or/1-4  6. medical education/ or medical residency/  7. medical students/  8. ((medic* adj3 (education or training or school* or curricul* or teaching or program*)) or ((resident* or "medical student*" or "medical trainee*") adj3 (education or training or teaching or learning)) or residency or "fellowship program*").ti,ab,id.  9. or/6-8  10. and/5,9  11. limit 10 to ("0100 journal" or "0110 peer-reviewed journal" or "0120 non-peer-reviewed journal" or "0130 peer-reviewed status unknown") |
| EBM Reviews; k= 0 | 1. ("artificial intelligence" or AI or Chatbot* or (conversational adj3 (robot* or agent*)) or (digital adj3 agent*) or ChatGPT or Gemini).ti,ab.  2. ((medic* adj3 (education or training or school* or curricul* or teaching or program*)) or ((resident* or "medical student*" or "medical trainee*") adj3 (education or training or teaching or learning)) or residency or "fellowship program*").ti,ab.  3. and/1-2 |
| Google Scholar; k=237 | ("artificial intelligence" OR AI OR Chatbot OR "conversational robot" OR "conversational agent" OR "digital agent" OR ChatGPT OR Gemini)  AND  (medical education OR medical training OR medical school OR medical curriculum OR medical teaching OR medical program OR residency OR fellowship program OR medical students OR medical trainees OR resident education OR student learning) |
