# Supplementary material for: Applications of Artificial Intelligence in Psychiatry and Psychology Education: Scoping Review
Source: JMIR Med Educ. 2025 Jul 28;11:e75238. doi: 10.2196/75238 (PMC12340458; doi:10.2196/75238)
Supplement: Multimedia Appendix 3 [file mededu_v11i1e75238_app3.docx]

**Multimedia Appendix 3.** Scoping review study selection detailed results.

This supplementary material has been provided by the authors to give readers additional information about their work.

**Table S1.** Scoping review study selection detailed results.

| **Authors** | **Population** | **Use of AI** | **Category** | **Main outcomes** | **Facilitators** | **Barriers** |
| --- | --- | --- | --- | --- | --- | --- |
| Anzia [1] | Psychiatrists and medical professionals engaged in continuing professional development (CPD) and certification processes | AI as a possible tool for clinical skills assessment (potential future use of AI in assessing clinical skills and enhancing continuing professional development for physicians) | Professional Development and Assessment | Transition from high-stakes examinations to longitudinal and interactive assessment models. Adoption of pilot programs such as the American Board of Psychiatry and Neurology (ABPN) Pilot Program, which provides flexible and relevant certification pathways. Evidence and support for modern adult learning principles in medical education and CPD. | Implementation of longitudinal assessment methods that align with adult learning principles. Introduction of article-based assessments that are flexible and relevant to practice. Use of innovative learning technologies (e.g., AI and digital tools) to improve assessment and engagement. | 1) There is limited evidence that continuing certification directly improves clinical practice or patient outcomes. 2) Physician concerns: Time, cost, burnout, and personal/professional balance issues related to maintenance of certification (MOC). 3) Bias in self-sssessment: Physicians often overestimate their competence, highlighting the importance of external assessments. 4) Negative stigma toward continuing certification requirements and their perceived burden. 5) While the article highlights interest in AI, it does not indicate any full-scale AI programs currently in place for board certification or residency |
| Banerjee et al [2] | 210 trainee doctors (Response rate 72%; 47% female) | AI technologies are being developed that are capable of automating many tasks typically performed by doctors as part of their clinical training such as decision support systems for triage, natural language processing for documentation, etc. | Clinical Decision Support, Administrative & Research Assistance, Natural Language Processing Applications | There was agreement that AI would have an overall positive impact on training and education. This agreement was sustained for ‘research and audit skills’ and ‘curriculum mapping’. Conversely, respondents tended towards disagreement for the domains of ‘clinical judgement/decision making’ (p = 0.12) and strong disagreement for the domain ‘clinical skills’ (p < 0.01). | Trainees agreed that AI would reduce clinical workload (62%) and improve training in research and audit skills (68%). | However, lower proportions agreed that it would improve training in clinical judgement and practical skills. The majority reported insufficient AI training in their current curricula (92%), and supported more formal AI training (81%). |
| Spallek et al [3] | Users of mental health and substance use educational materials, including educators, researchers, and the general public. Specific emphasis on audiences such as youth, parents, teachers, health professionals, and substance use clients | ChatGPT-4 utilization in generating mental health and substance use educational materials. The study evaluates the model's ability to: 1) Answer direct user queries, 2) Create and edit educational materials. 3) Customize content. 4) Adhere to communication guidelines. It then examines the outputs' quality and accessibility. | Educational Content Creation & Enhancement, Natural Language Processing Application, Program/Policy Development | 1) GPT-4 was able to generate well-structured and accessible health education materials, especially when detailed prompts were used. It could simplify complex topics and adapt content to different audiences with some degree of cultural sensitivity. 2) Challenges that were identified: GPT-4 struggled with accuracy, consistency in following communication guidelines, and cultural inclusivity. It was unsuitable for direct health advice due to risks of misinformation and limited depth. 3) Carefully designed prompts significantly improve the quality and relevance of AI-generated content. 4) Necessity of human oversight: While GPT-4 can assist in developing educational materials, human oversight and expert review remain essential for accuracy and reliability. | Facilitator of AI uses: 1) Tools like GPT-4 increase efficency of content creation, making it faster and more accessible for educators. 2) Audience-specific communication: AI adapts its language to suit different groups, ensuring communication is both culturally relevant and audience-centered. 3) AI-generated content is well-structured and high-quality (user-friendly content), reducing the need for extensive manual revisions. 4) Simplifying complex information. 5) Customizable content: By adjusting prompts, users can refine outputs to reduce bias, encourage positive behaviors, or meet specific needs. 6) Quick drafting of materials: AI enables the rapid creation of initial drafts for educational content, which can then be refined by experts. Study's methodology: 1) Structured approach: Prompts were carefully designed to ensure GPT-4 adhered to communication standards, including avoiding stigmatizing language. 2) The study ensured that outputs reflected recognized guidelines in mental health education. 3) AI-generated content was reviewed multiple times to improve its quality and relevance. 4) Clear criteria were used to evaluate the outputs (e.g. readability and adherence to guidelines). 5) GPT-4 was assessed in different educational contexts to demonstrate its flexibility. 6) Outputs were tailored to be sensitive to the needs of specific groups, such as parents or youth. 7) Transparent methods: The study openly described its methodology and limitations to allow for replication and improvement. | Limitations: 1) Need for extensive prompting to ensure culturally sensitive and non-stigmatizing communication. 2) Insufficient formal education for users, leading to potential misuse of AI technologies. 3) Potential biases in AI outputs and inadequate guidance on managing ethical dilemmas. 4) Outputs occasionally contain inaccurate or incomplete information. 5) AI-tools can show inconsistent adherence to evidence-based guidelines and communication standards. 6) AI-tools have Limited training data for minority populations and cultural sensitivities : some outputs of AI-tools can include stigmatizing language or lack cultural relevance. 7) Generated materials often exceed recommended readability levels (grade 7-10). 8) Need for extensive prompting to ensure culturally sensitive and non-stigmatizing communication. |
| Mangold and Ream [4] | Medical students, residency applicants, faculty members and program directors involved in the graduate medical education (GME) application process. | 1) Personal statements: Applicants use AI tools like ChatGPT for drafting and editing personal statements, improving grammar, brainstorming, and enhancing clarity, especially for non-native English speakers. 2) Letters of recommendation: AI assists faculty in generating LOR drafts to save time, reduce biased language, and improve writing efficiency. 3) Applications screening: AI tools can help program directors by filtering applications based on predefined criteria, potentially mitigating human biases. 4) AI is proposed as a tool to help programs generate guidelines for AI use in applications. | Student/Applicant Support, Program/Policy Development | Advantages: AI tools improve accessibility, efficiency, and equity in application processes by providing affordable and easy-to-use assistance. Can reduce biased language in LORs and streamline application reviews. Concerns: AI-generated content often lacks authenticity, nuance, and personal details critical for personal statements and LORs. The potential for AI to perpetuate biases present in its training data. Difficulty in detecting AI-generated content and ensuring authenticity in application materials. | Prompt engineering: Structured prompts improve the quality and relevance of AI-generated outputs. Human oversight: Heavy editing by applicants or faculty can refine AI-generated drafts to meet expectations. AI detection Software: Tools are available but have limitations, particularly with non-native English speakers’ text | 1) AI-written materials lack the unique voice and personal connection that human authors provide. 2) AI training data can embed and propagate existing biases (e.g., gender or cultural). 3) AI-detection software has high false-positive rates and is unreliable, especially for diverse linguistic backgrounds. 4) Unclear policies: Current policies and guidelines do not adequately address the complexities of AI use in the GME application process. 5) The ethical concerns of disclosing AI use in application materials remain a debated issue, with limited enforceable guidelines. |
| Blease et al [5] | 37 out of 120 students enrolled in a two-year Master's program in clinical psychology and psychotherapy at University of Basel, Switzerland (response rate : 31%). Participation was voluntary. NB: Among the respondents, 73% (n=27) planned to pursue a career in mental health, and 97% indicated familiarity with the term "machine learning". | 1) Psychotherapy apps & tools' ability to track mental health symptoms in real-time. 3) AI's role in providing therapies like CBT and ACT. 4) AI's potential to improve mental health diagnosis (decision support). 5) AI's capacity to tailor treatments, including medication selection. 6) AI's role in identifying high-risk patients and supporting early intervention (preventative care). 7) AI as a tool to assist therapists by handling administrative tasks. | Clinical Decision Support, Therapeutic Tools & Mental Health Monitoring, Administrative & Research Assistance | 1) 97% of participants were aware of ML and 78% had knowledge of big data analytics. 2) Postgraduate students reported minimal exposure to AI/ML within their degree (they estimated that only 0.52% of their program would be dedicated to AI/ML education). 3) 46% of the participants stated that they intented to learn more about AI/ML uses that pertain to mental health care. 4) Students "moderately agreed" (median=4) that AI/ML ought to be included in clinical psychology and psychotherapy education (on a 5-point Likert scale). 5) The only significant positive correlation observed was between respondents' attitudes toward incorporating AI/ML into education and the number of hours they spent receiving relevant training. Students with more hours of AI/ML education gave higher ratings on the five-point Likert scale (r=0.34, p=0.038). 6) Qualitative analysis of students' comments identified 4 key themes regarding the impact of AI/ML on the psychology/ psychotherapy provision of care: "changes in the quality and understanding of psychotherapy care", effects on patient-therapist interactions, implications for the psychotherapy profession, issues pertaining to data management and ethics. Conclusions: The study revealed that postgraduate clinical psychology students expressed diverse opinions but had limited official training regarding the potential impact of AI/ML-based tools on psychotherapy. The findings shed light on the necessity to explore ways to adapt curricula to better educate clinical mental health trainees about the role of AI/ML in this field. | Facilitator of AI uses: 1) AI can improve accessibility to mental health services, which could have a secondary positive impact on public health. 2) AI/ML can contribute to identifying "new patterns" and advancing research on the mechanisms and underlying causes of mental illness. 3) AI/ML's potential to customize mental healthcare, such as adapting psychopharmacological treatments (e.g., determining the most appropriate SSRI for a patient). 4) Increased diagnostic accuracy and "more targeted treatments". 5) AI tools can play a significant role in predicting mental health risks, identifying high-risk patients, and enabling early detection. 6) AI/ML tools could reduce the administrative workload of therapists, enabling them to focus on critical aspects of care. Facilitators derived from the methodology: 1) Use of a convenience sample: the study targeted postgraduate clinical psychology students enrolled in a master's program at a leading European university, ensuring a well-defined and accessible population. 2) The survey was conducted online using user-friendly software (Jisc), facilitating widespread access during the COVID-19 pandemic when face-to-face interactions were limited. 3) Mixed-methods approach: Combining quantitative and qualitative analyses allowed a good assessment of student attitudes. 4) Pre-testing of the survery instrument via psychology students outside the study cohort, to ensure face validity and feasibility, enhancing the quality and reliability of the responses. 5) Expert consultation: Academic informaticians from Harvard Medical School and psychotherapists from the University of Basel contributed to the survey design, ensuring its relevance to educational and clinical contexts. 6) Pseudonymized and voluntary participation increases honest responses and participation rates. | Limitations: 1) AI/ML tools can sometimes produce inaccurate results, and the algorithms driving them may be difficult for humans to fully understand. 2) Innovations could create a misleading perception of how easily care can be accessed and utilized. 3) A fundamental aspect of psychotherapy is the patient-therapist relationship (human qualities like empathy), which AI cannot replicate. 4) Concerns regarding violation of privacy, the security of patient data and its potential misuse (risk of third-party exploitation or hacking). 5) Ethical challenges (e.g. "digital divide, patient digital literacy in using apps" and algorithmic biases in AI design) were overlooked. 6) The regulation, approval processes, and evidence-based data pertaining to existing mental health apps were barely discussed. This lack of attention is concerning, especially as the digital app market expands rapidly, offering potential benefits but also notable risks. 7) Limited student awareness: Students demonstrated minimal formal training in AI/ML topics, which may hinder their ability to critically evaluate or guide the use of these tools in practice. 8) Some students expressed the belief that AI/ML would not influence psychotherapy either in the near or distant future. Nearly half of the respondents indicated uncertainty or a lack of intention to pursue further learning on AI/ML. |
| Hudon et al [6] | 102 respondents residents and clinician-educators in the field of psychiatry (45 (44.1%) identified as medical residents in psychiatry, 2 (2%) identified as teaching psychiatrists with less than 5 years of experience, 16 (15.7%) identified as teaching psychiatrists with between 6 and 10 years of experience, and 39 (38.2%) identified as teaching psychiatrists with more than 10 years of experience.) | AI (ChatGPT) generating script concordance texts (SCTs) to be used to promote a higher level of clinical reasoning | Educational Content Creation & Enhancement | The aim of this study was to compare SCTs created by ChatGPT to SCTs produced by clinical specialists on the scenario (stem), clinical questions, and expert opinions. There were no significant distinctions between the SCTs generated by ChatGPT as compared to those developed by experts in the field for the evaluated components. | Almost all respondents (32/39, 82%) mentioned that the SCTs were using typical clinical signs and symptoms reported in the DSM-5. Some (5/39, 13%) indicated that the SCTs were very well nuanced. | Most respondents (29/39, 74%) reported the SCTs generated by ChatGPT as caricatural or stereotypical clinical presentations as observed in textbooks with little regard to atypical presentations. A total of 7 (18%) respondents indicated that the SCTs generated by ChatGPT were too simple, as they tended to include additional information that were too trivial when attempting to challenge the responder’s clinical reasoning. |
| Manjunatha et al [7] | 25 In Person-On Consultation Training (IP-OCT) sessions for 35 Primary Care Doctors (PCDs) matching 100 hours. 61 total Tele-On-ConsultationTraining (Tele-OCT) sessions were conducted (59 Primary Care Doctors (PCDs) uderwent at least one session, and 23 completed the 3 sessions) matching 175h of Tele-OCT were conducted. Over 12 000 patients were seen in the first 9 months of implementation. | Potential use of AI in the future to design language compatible AI-based Tele-OCT modules to expand the program. | Program/Policy Development | This paper provides an overview of the 5 modules of the Primary Care Psychiatry Program (PCPP) recently implemented in India, with a focus on On-Consultation-Training. Results through verbal feedback from the PCDs. IP-OCT: acceptable, realistic and feasible model that could be replicated in their practice and the translational quotient was thought to be higher than with standard training. Tele-OCT: more implementable at a larger scale and higher acceptability. No data was provided in the article. | OCT provides direct skill-transfer without disrupting the PCDs' clinical work. The patient population matches the reality of primary care work. Tele-OCT was more implementable at a larger scale than IP-OCT by adressing the challenge of traveling trainer-psychiatrists and reducing costs. Higher acceptability was found among the PCDs receiving the training in Tele-OCT. | There are limitations related to finding enough psychiatrists to train the PCDs in the program as well as cost-related limitations regarding the salary of these psychiatrists. The program also relies on available laptops and adequate internet connectivity in the healthcare centers. |
| Gratzer and Goldbloom [8] | Not applicable | Chatbots in mental health, E-therapy purposes. | Therapeutic Tools & Mental Health Monitoring | 1) E-therapies, including those powered by AI, are becoming a significant component of mental health care. 2) Internet-provided cognitive behavioral therapy (iCBT) and AI-powered apps (chatbots) show promise for improving access to care and delivering evidence-based interventions. Moderate to large effects have been reported for iCBT in treating conditions like panic disorder, social anxiety disorder, generalized anxiety disorder, PTSD, and major depression. AI tools like chatbots are still in early stages of research but show positive potential for user engagement and experience. | 1) The widespread availability of smartphones and internet connectivity facilitates the use of apps and web-based interventions, making e-therapy accessible to a broad population. Patients can access therapy tools anytime, breaking traditional barriers related to geography or scheduling. Many patients, particularly younger demographics, are already engaging with mental health apps. For instance, according to a referenced study, 80% of patients under 25 at a Boston clinic had downloaded a mental health app. 2) AI-powered tools can mimic human-like behaviors and offer task-oriented frameworks with evolving dialogues, making them suitable for therapeutic purposes. | 1) Challenges of E-therapies: High dropout rates for non-guided therapies highlight the importance of human involvement or structured guidance. Many apps lack quality and transparency, with only a fraction meeting basic clinical standards. 2) Psychiatry training needs to adapt to incorporate e-therapies, requiring learners to become proficient in: exploring and evaluating apps, guiding patients in selecting suitable e-therapy tools, delivering therapy via non-traditional mediums (e.g. telepsychiatry, apps). 3) Concerns about privacy, data sharing, and consent. |
| López-Ojeda and Hurley [9] | Individuals impacted by neurological disorders (such as Parkinson’s disease, Alzheimer’s disease, epilepsy), by substance use disorders and by mental health issues (such as depression, anxiety, and suicidality). It also encompasses clinicians, medical students, and researchers using AI-tools in psychiatry and clinical neurosciences. | 1) Diagnostic support using AI algorithms for analysis of neuroimaging. 2) Prognosis and monitoring of neurodegenerative diseases, tailoring treatments using digital phenotyping. 3) Chatbots (e.g. Leora) for depression, anxiety self-assessment and support. 4) Natural language processing (NLP) algorithms for suicide risk detection based on online interactions. 5) AI-assisted learning (e.g. ChatGPT) for medical education and licensing exam preparation. 6) Supporting medical research by automating literature review and article drafting. 7) Prediction of risks and outcomes of substance use disorders using machine learning models. | Clinical Decision Support, Educational Content Creation & Enhancement, Therapeutic Tools & Mental Health Monitoring, Administrative & Research Assistance, Natural Language Processing Applications | 1) AI and large language models (LLMs) are revolutionizing psychiatry and clinical neurosciences, aiding in early disease detection, personalized treatments, and real-time monitoring. These technologies are used to manage neurological conditions like Alzheimer’s, Parkinson’s, and epilepsy, as well as mental health challenges such as depression and anxiety. 2) Chatbots, including Woebot and Leora, improve access to mental health support and play a role in suicide prevention. 3) AI tools assist in educating individuals about substance use and predicting associated risks. 4) In medical education, LLMs like ChatGPT demonstrate potential as learning aids, achieving scores similar to medical students on licensing exams.These tools streamline research processes, enhancing productivity in scientific writing and evidence review. | 1) Mental health chatbots (e.g. Woebot) increase accessibiliy to support for anxiety, depression, and substance use disorders. 2) AI-powered wearable devices offer real-time feedback for treatment adjustments. 3) Tools like ChatGPT simulate exams and support learning. 4) Advanced models (e.g. GPT-4) process vast information for education, diagnostics, and research. | 1) In research and education, AI tools like ChatGPT raise concerns about plagiarism, originality, and ethical authorship, as they may generate content that lacks proper citation or critical evaluation. 2) While AI can process large datasets, it often fails to generate highly individualized or context-specific insights, particularly when faced with unique or rare cases. 3) AI tools (including LLMs) often lack the ability to perform clinical reasoning, logical analysis and display empathy, especially if clinicians over-rely on them. 4) AI outputs can be influenced by biases stemming from the data they are trained on, leading to inequitable outcomes or misdiagnoses, particularly in underrepresented patient populations, |
| Tortora [10] | Not applicable | Discriminative AI in forensic psychiatry is violence and recividism risk assessment.  Generative AI carry the promise not only to influence traditional discriminative AI applications, like risk assessment and personalised treatment design but also to create new opportunities in areas previously underexplored, such as training and education. | Clinical Decision Support | Discrimination AI and Generative AI could aid in advancing the field of forensic psychiatry in different aspects, however this comes with new sets of challenges and ethical concerns. | GenAI technologies, such as Generative Adversarial Networks (GANs), transformer-based models, and others, enable the creation of realistic data and integration of unstructured content across various formats. This advancement is poised to redefine traditional practices like risk assessment, diagnostic support, and treatment planning, while also opening new avenues in training and education. | Risk of AI include the risk of perpetuating societal biases, exacerbating discrimination, and creating unequal access to resources. There are also worries about increased surveillance of marginalized groups. The authors stress the need for interdisciplinary collaboration and thorough evaluations to ensure responsible adoption of GenAI, particularly in contexts where decisions can significantly impact individuals' lives |

**Table S2.** Summary of the quality assessment.

| **Study** | **Type of Article** | **Assessment Tool Used** | **Quality Assessment Summary** |
| --- | --- | --- | --- |
| Hudon et al [6] | Mixed Methods | MMAT (2018) | High quality; clear mixed methods integration, sound methodology, ethical compliance, and transparent limitations. |
| Blease et al [5] | Mixed Methods | MMAT (2018) | Moderate to high quality; coherent data collection and analysis, but integration of methods could be more explicit. |
| Spallek et al [3] | Viewpoint / Case Study | AACODS Checklist | Conceptually robust; strong authority and relevance, but lacks empirical validation and formal methodology. |
| Tortora [10] | Conceptual / Theoretical | AACODS Checklist | Strong in authority, coverage, and significance; article provides an in-depth ethical analysis of generative AI in forensic psychiatry. However, it lacks empirical validation and methodological transparency. |
| Banerjee et al [2] | Quantitative (Survey) | JBI Checklist for Analytical Cross-Sectional Studies | Moderate quality; clear aim and relevant findings. However, limited detail on sampling strategy, potential bias, and validation of the survey instrument. |
| Mangold and Ream [4] | Perspective / Commentary | AACODS Checklist | Strong relevance and authority; timely discussion of AI in application processes. However, lacks systematic data collection or validation, limiting empirical robustness. |
| Anzia [1] | Opinion / Commentary | AACODS Checklist | Focused and well-argued; reflects expert insights on lifelong learning in psychiatry. Nonetheless, it lacks data, structured methodology, and generalizability. |
| López-Ojeda and Hurley [9] | Conceptual / Narrative Review | AACODS Checklist | Comprehensive coverage and technically sound overview of AI/LLM in psychiatry. No primary data or systematic review criteria applied. |
| Manjunatha et al [7] | Descriptive / Implementation Report | JBI Checklist for Analytical Cross-Sectional Studies (adapted) | Innovative and practical; offers useful insights for digital psychiatric education. Limited methodological detail and lacks outcome data validation. |
| Gratzer and Goldbloom [8] | Perspective / Commentary | AACODS Checklist | Well-articulated discussion on e-therapy and apps. Authoritative and relevant, but informal design with no data validation. |

## References

1. Anzia JM. Lifelong Learning in Psychiatry and the Role of Certification. Psychiatr Clin North Am. 2021;44(2):309-316. doi:10.1016/j.psc.2021.03.001
2. Banerjee M, Chiew D, Patel KT, et al. The impact of artificial intelligence on clinical education: perceptions of postgraduate trainee doctors in London (UK) and recommendations for trainers. BMC Med Educ. 2021;21(1):429. Published 2021 Aug 14. doi:10.1186/s12909-021-02870-x
3. Spallek S, Birrell L, Kershaw S, Devine EK, Thornton L. Can we use ChatGPT for Mental Health and Substance Use Education? Examining Its Quality and Potential Harms. JMIR Med Educ. 2023;9:e51243. Published 2023 Nov 30. doi:10.2196/51243
4. Mangold S, Ream M. Artificial Intelligence in Graduate Medical Education Applications. J Grad Med Educ. 2024;16(2):115-118. doi:10.4300/JGME-D-23-00510.1
5. Blease C, Kharko A, Annoni M, Gaab J, Locher C. Machine Learning in Clinical Psychology and Psychotherapy Education: A Mixed Methods Pilot Survey of Postgraduate Students at a Swiss University. Front Public Health. 2021;9:623088. Published 2021 Apr 9. doi:10.3389/fpubh.2021.623088
6. Hudon A, Kiepura B, Pelletier M, Phan V. Using ChatGPT in Psychiatry to Design Script Concordance Tests in Undergraduate Medical Education: Mixed Methods Study. JMIR Med Educ. 2024;10:e54067. Published 2024 Apr 4. doi:10.2196/54067
7. Manjunatha N, Kumar CN, Math SB, Thirthalli J. Designing and implementing an innovative digitally driven primary care psychiatry program in India. Indian J Psychiatry. 2018;60(2):236-244. doi:10.4103/psychiatry.IndianJPsychiatry_214_18
8. Gratzer D, Goldbloom D. Therapy and E-therapy-Preparing Future Psychiatrists in the Era of Apps and Chatbots. Acad Psychiatry. 2020;44(2):231-234. doi:10.1007/s40596-019-01170-3
9. López-Ojeda W, Hurley RA. Medical Metaverse, Part 2: Artificial Intelligence Algorithms and Large Language Models in Psychiatry and Clinical Neurosciences. J Neuropsychiatry Clin Neurosci. 2023;35(4):316-320. doi:10.1176/appi.neuropsych.20230117
10. Tortora L. Beyond Discrimination: Generative AI Applications and Ethical Challenges in Forensic Psychiatry. Front Psychiatry. 2024;15:1346059. Published 2024 Mar 8. doi:10.3389/fpsyt.2024.1346059
